# The ectopic expression of *Arabidopsis* glucosyltransferase UGT74D1 affects leaf positioning through modulating indole-3-acetic acid homeostasis

**DOI:** 10.1038/s41598-021-81016-x

**Published:** 2021-01-13

**Authors:** Shanghui Jin, Bingkai Hou, Guizhi Zhang

**Affiliations:** 1 College of Life Sciences, Qingdao Agricultural University, Qingdao, 266109 China; 2 School of Pharmacy, College of Pharmacy, Linyi University, Shuangling Road, Lanshan District, Linyi, 276000 China; 3 The Key Laboratory of Plant Development and Environment Adaptation Biology, Ministry of Education, College of Life Sciences, Shandong University, Qingdao, 266237 China

**Keywords:** Molecular biology, Plant sciences

## Abstract

Leaf angle is an important agronomic trait affecting photosynthesis efficiency and crop yield. Although the mechanisms involved in the leaf angle control are intensively studied in monocots, factors contribute to the leaf angle in dicots are largely unknown. In this article, we explored the physiological roles of an *Arabidopsis* glucosyltransferase, UGT74D1, which have been proved to be indole-3-acetic acid (IAA) glucosyltransferase in vitro. We found that UGT74D1 possessed the enzymatic activity toward IAA glucosylation in vivo and its expression was induced by auxins. The ectopically expressed *UGT74D1* obviously reduced the leaf angle with an altered IAA level, auxin distribution and cell size in leaf tissues. The expression of several key genes involved in the leaf shaping and leaf positioning, including *PHYTOCHROME KINASE SUBSTRATE* (*PKS*) genes and *TEOSINTE BRANCHED1*, *CYCLOIDEA*, and *PCF* (*TCP*) genes, were dramatically changed by ectopic expression of *UGT74D1*. In addition, clear transcription changes of *YUCCA* genes and other auxin related genes can be observed in overexpression lines. Taken together, our data indicate that glucosyltransferase UGT74D1 could affect leaf positioning through modulating auxin homeostasis and regulating transcription of *PKS* and *TCP* genes, suggesting a potential new role of UGT74D1 in regulation of leaf angle in dicot *Arabidopsis*.

## Introduction

Auxin, primarily indole-3-acetic acid (IAA), is an endogenous plant hormone that plays a crucial role in plant growth and development. It contributes to many aspects in vivo such as plant organ development, plant geotropic and phototropic responses, formation and differentiation of vasculature, apical dominance, senescence and responses to environmental stresses^1–5^. Since many aspects of auxin action strictly depend on its differential concentration and distribution within plant tissues, and higher concentrations of auxin might often produce inhibitory effects, so the optimum endogenous level must be strictly controlled by biosynthesis, degradation, conjugation and polar transport^6,7^.

Glucose conjugation is considered to maintain metabolic balance for auxin, since the substrate chemical properties such as solubility, bioactivity and transport are affected by glycosylation^8,9^. It has been reported that auxin glucose conjugates have been isolated from plants, suggesting the existence of auxin glycosyltransferases in plants^10,11^. IAGLU was first identified as an IAA glucosyltransferase in *Zea mays*, which could catalyze the formation of IAA glucose ester from IAA and glucose^12^. In addition, two auxin glucosyltransferases including UGT84B1, UGT74E2 from *Arabidopsis* were also isolated and identified, but their glucosylating activity toward IAA and indole-3-butyric acid (IBA) differs a lot. It was indicated that UGT84B1 has high in vitro catalytic specificity to both IAA and IBA, and by contrast, UGT74E2 prefers IBA to IAA^13–15^. Transgenic plants overexpressing these genes in *Arabidopsis* showed obvious growth deficiency phenotypes. For instance, the *UGT84B1* and *UGT74E2* overexpression lines exhibited dwarf stature, increased shoot branches and compressed rosette^14,15^. Meanwhile, overexpression of *UGT84B1* resulted in the curly leaves in contrast to wild type^14^. Another *Arabidopsis* gene UGT84A2 was an indole-3-butyric acid glucosyltransferase and involved in delayed flowering^16^, suggesting that the auxin glucosylation might play a significant role in the regulation of plant reproductive development.

The leaf positioning is an important agronomic trait which is closely related to the agricultural yields and architecture. Most researches on leaf positioning were carried out in monocots (especially rice) and indicated that leaf positioning was controlled by the phytohormone brassinosteroid (BR) related signaling pathway^17–21^. Recently, Zhao and colleagues reported a mutant of IAA-amido synthetase (*lc1-D*) from rice^22^. This mutant showed exaggerated leaf angles because the cells at the lamina joint were stimulated to elongate. Further study showed that *lc1-D* mutant was particularly sensitive to exogenous BR and had significantly reduced the expression of BR biosynthetic genes, suggesting that LC1 may regulate rice leaf positioning through the interaction between auxin and BR^22^. For dicots, several researches showed that the leaf positioning was mostly related to light signaling and auxin signaling^23–26^. For example, the *Arabidopsis PHYTOCHROME KINASE SUBSTRATE* (*PKS*) family and *TEOSINTE BRANCHED1*/*CYCLOIDEA*/*PCF* (*TCP*) family were demonstrated to be required for the regulation of leaf development and leaf positioning^26–28^. Auxin biosynthesis and polar transport were also demonstrated to be involved in the leaf positioning control^23–25^. As mentioned above, auxin glucosylation plays an important role in the regulation of plant growth and development. However, whether the auxin glucosylation plays a role in the case of leaf positioning is largely unknown.

Previously, we identified the glycostransferase UGT74D1, which was found to have the glucose conjugating activity toward both IAA and its precursor IBA, with a little preference toward the latter^29^. In this study, we characterized the growth responses of ectopically expressed *UGT74D1* transgenic plants and mutants. It was found that the increased UGT74D1 activity substantially altered auxin distribution in leaf primordial and resulted in accumulation of free IAA in leaves, which then dramatically stimulated cell elongation and led to BR independent change of leaf positioning, possibly by a feedback transcription regulation of PKS and TCP factors. Our work provides evidences for the link between auxin glucosylation, auxin homeostasis and leaf positioning in dicots, highlighting a distinct role of UGT74D1 from other auxin glycosyltransferases identified so far.

## Results

### Expression of *UGT74D1* was developmentally regulated and induced by auxins

**Figure 1:**
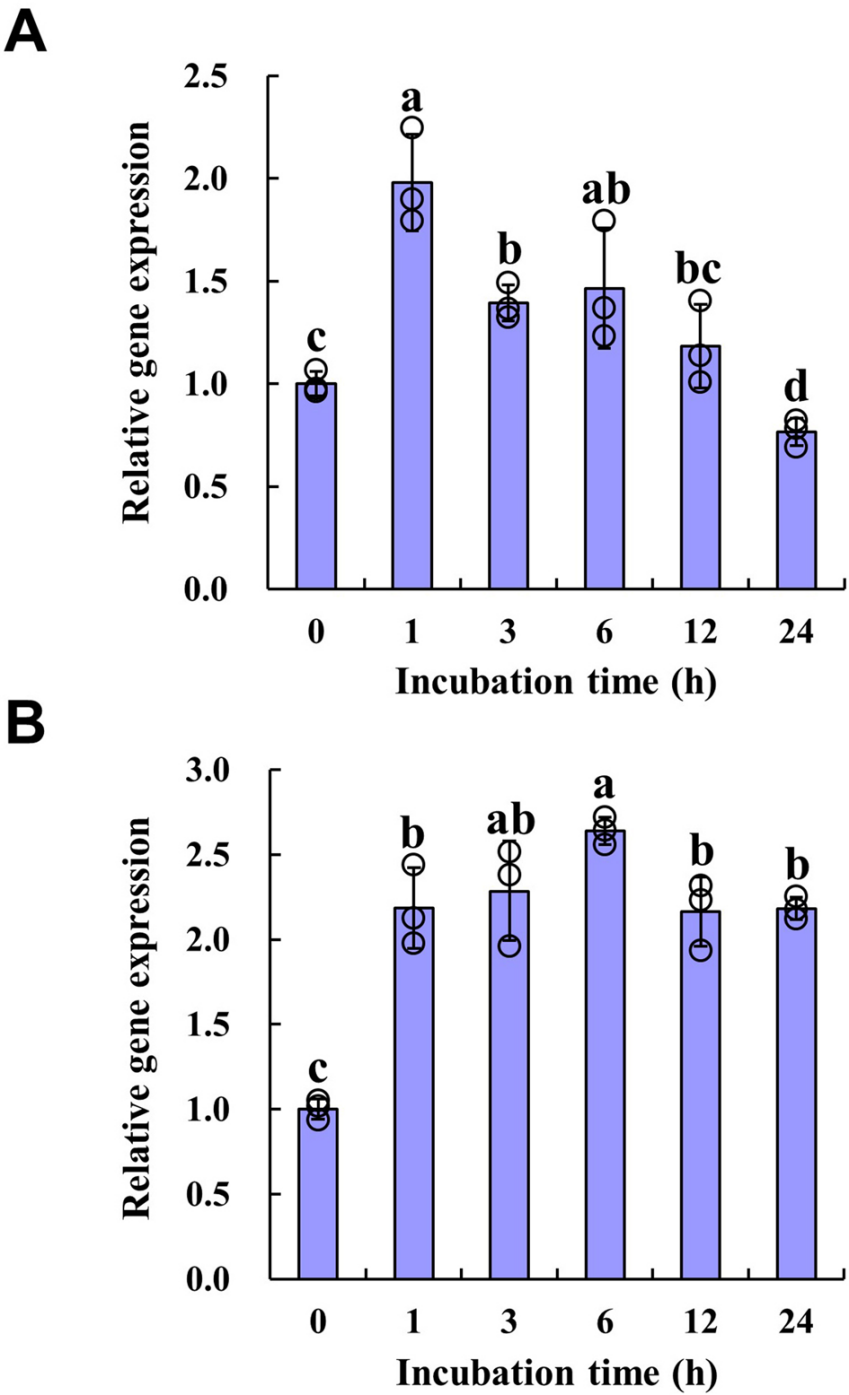
The induced expression of *UGT74D1* gene by 10 μM IAA (**A**) or 10 μM IBA (**B**). The relative transcript level was normalized to the transcript abundance of *Actin 2* gene. The statistical significance of the difference was confirmed by ANOVA at α = 0.05 level. Error bars indicate SD from triplicate experiments.

To explore the physiological role of UGT74D1, we first investigated whether the expression of *UGT74D1* gene is responsive to auxins, since UGT74D1 recombinant protein can catalyze the glucose conjugation of auxins as indicated by our previous work^29^. Two types of natural auxins, IAA and IBA, were used in this experiment. The results of qRT-PCR analysis indicated that both IAA and IBA could induce the expression of *UGT74D1* within different treatment duration from 1 to 24 h (Fig. 1), suggesting that UGT74D1 might function in the auxin homeostasis.

**Figure 2:**
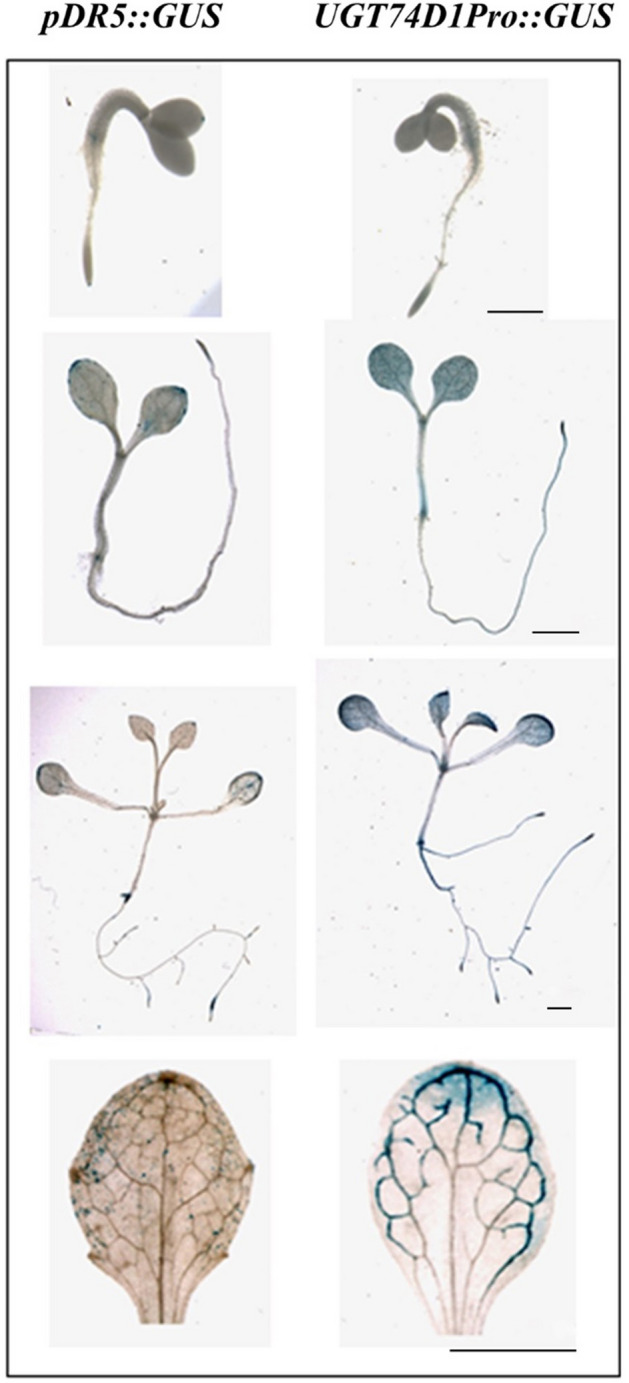
Localization of the auxin reporter *pDR5::GUS* activity and *UGT74D1pro::GUS* activity in cotyledon, hypocotyl, root, root apex, leaf and leaf edge. Scale bar = 1 mm.

In addition, the localization of auxin activity and *UGT74D1* expression in wild type plants was also investigated by *pDR5::GUS* and *UGT74D1 promoter::GUS* constructs. Interestingly, our analyses indicated that the localization of *pDR5::GU*S activity and *UGT74D1 promoter::GUS* activity was nearly overlapping at cotyledon, hypocotyl, root, root apex, leaf and leaf edge (Fig. 2). Thus, the coincidence among the zone of auxin activity and the mostly expressed site of *UGT74D1* gene was particularly meaningful for role of UGT74D1 exerted in planta, suggesting a relevance of the UGT74D1 expression in mediating auxin homeostasis.

**Figure 3:**
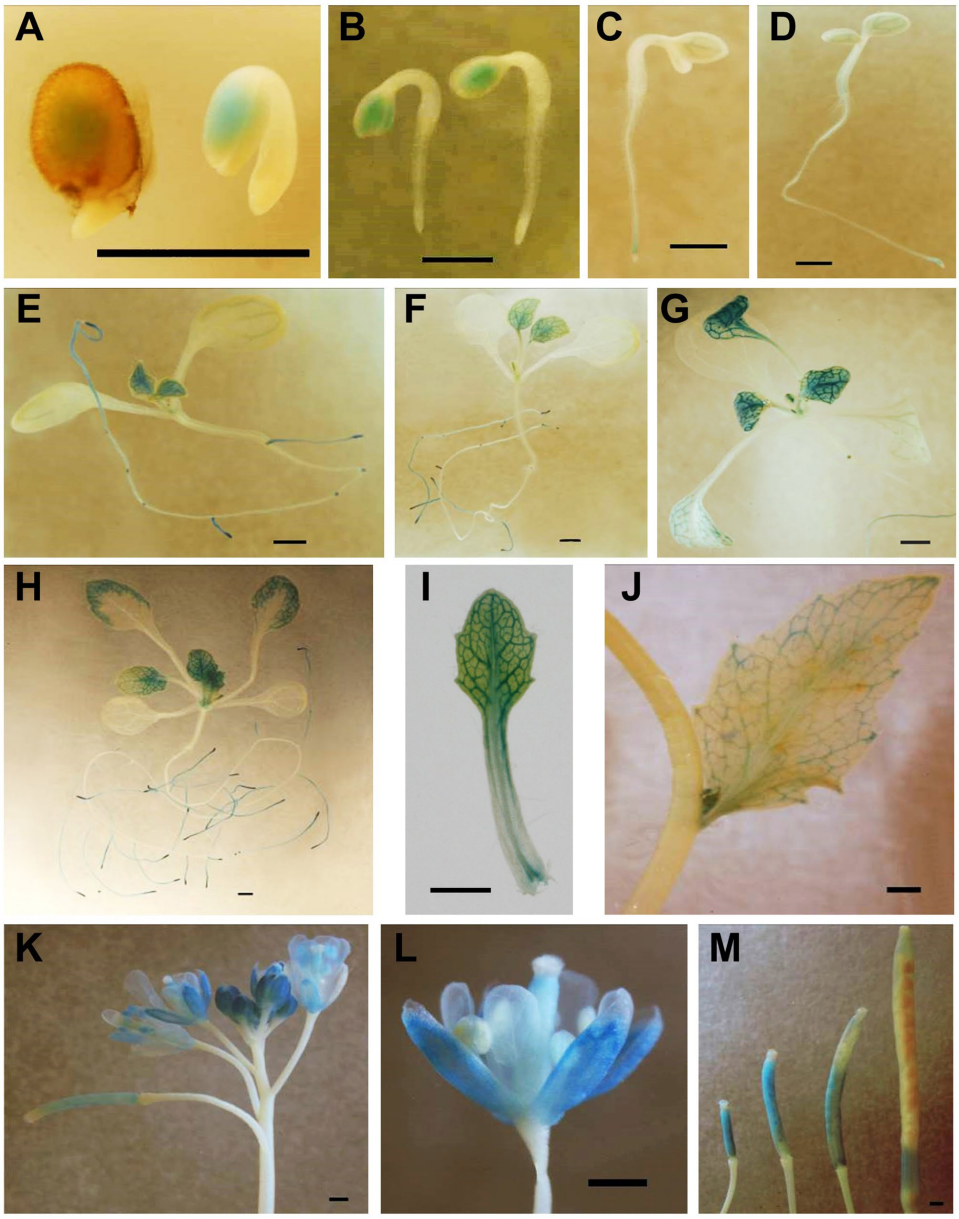
The expression pattern of *UGT74D1* gene reported by GUS expression. (**A**–**D**) *UGT74D1* expression was found mainly in the cotyledon for 1 to 4-day old seedlings. (**E**–**G**) *UGT74D1* expression was found mainly in whole young leaf and root tip. (**H**) *UGT74D1* expression was moved to leaf edge in mature leaf. (**I**) *UGT74D1* was expressed in young leaf petioles. (**J**–**M**) *UGT74D1* expression was found in veins of cauline leaf, flower and young silique. Scale bar = 1 mm.

Histochemical specific staining of GUS activity in the *UGT74D1 promoter::GUS* transgenic lines showed that *UGT74D1* was strongly expressed in cotyledons during early germination (Fig. 3A–D). In the subsequent vegetative growth stage, *UGT74D1* was expressed mainly in young leaves, leaf veins (Fig. 3E–H). Importantly, *UGT74D1* was strongly expressed in young leaf petioles (Fig. 3I), which implies the biological function of UGT74D1 in leaf petiole development. During the reproductive stage, *UGT74D1* was highly expressed in flowers, young siliques and veins of cauline leaves (Fig. 3J–M). These results indicate that the expression pattern of *UGT74D1* is spatio-temporally regulated.

### UGT74D1 was localized in nucleus and cytoplasm

**Figure 4:**
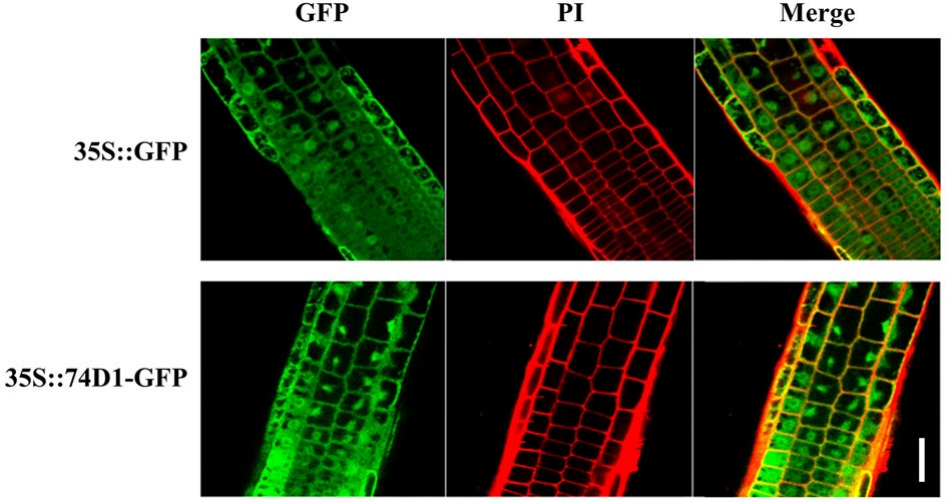
The subcellular localization of UGT74D1 protein. 35S::GFP construct was used as the control in this study. 35S::UGT74D1-GFP construct was used to produce the fusion protein of UGT74D1-GFP. PI: Propidium Iodide staining. Scale bar = 10 μm.

To investigate the subcellular localization of UGT74D1 protein, the plasmid *35S::74D1-GFP* was transformed into *Arabidopsis*. The roots of the 5-day-old transgenic seedlings were detected for getting the fluorescent images. The distribution of green fluorescence signals of the UGT74D1-GFP fusion protein was similar as that of the control GFP protein, indicating that UGT74D1 protein was localized in both nucleus and cytoplasm (Fig. 4). Similar subcellular localization patterns of other UGTs in plants have also been observed in nucleus and cytoplasm, including PpUGT85A2 glycosylates linalool^30^, UGT85A1 glycosylates zeatin^31^, UGT87A2 involved in flower development regulation^32^, and UGT73C6 glycosylates brassinosteroids^33^. UGTs may also play a role in the nucleus to control the stability of nuclear receptor ligands and protect nuclear components from toxins^34,35^.

### UGT74D1 possessed activity toward IAA *in planta*

To investigate whether UGT74D1 glucosyltransferase has activity toward IAA in planta, at least four overexpression lines (*74D1OE-11*, *-23*, *-24*, *-26*) and two independent T-DNA insertion mutants (*74d1ko-1*, Salk_004870; *74d1ko-2*, Salk_011286) were used in this study with the same line codes used previously^29^.

**Figure 5:**
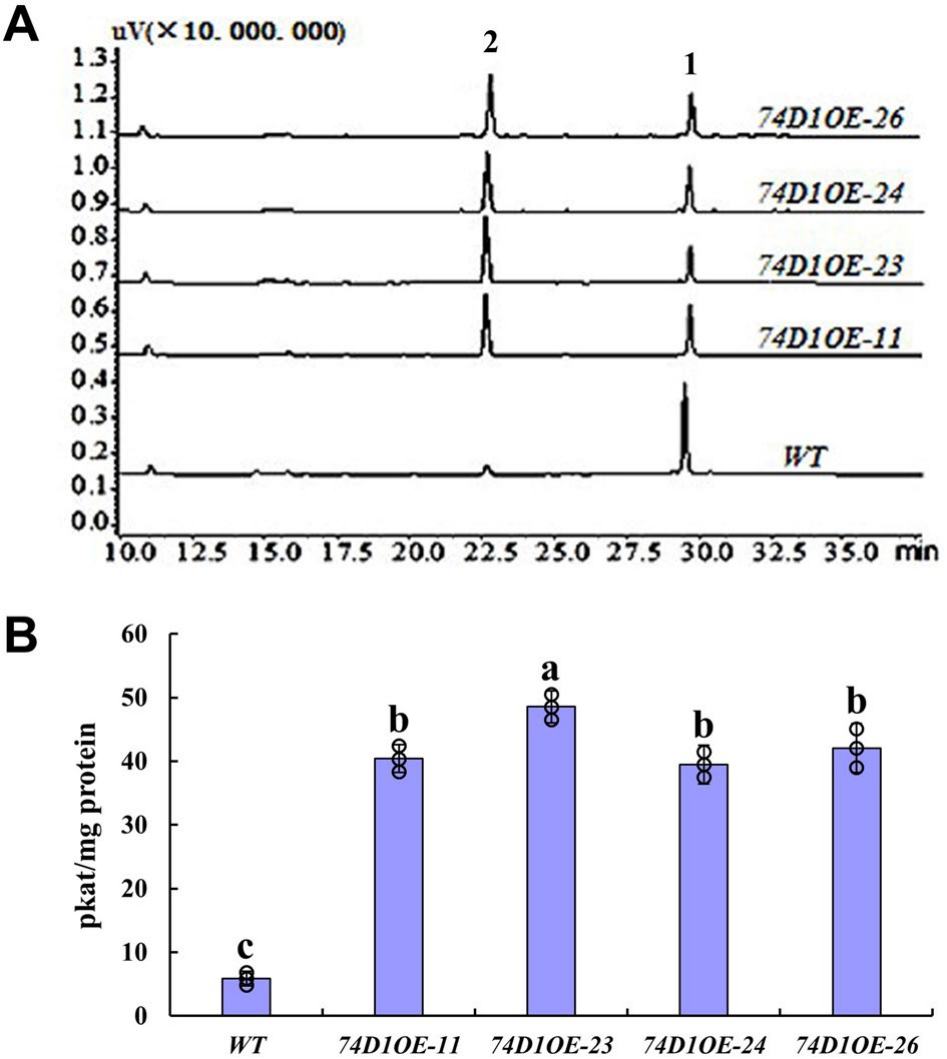
The glucosyltransferase activity of transgenic plants toward IAA. (**A**) HPLC analysis of glucosyltransferase activity of transgenic plants. 1, substrate IAA; 2, glucosyl ester of IAA. (**B**) Calculated activity value of glucosyltransferase activity of transgenic plants. The statistical significance of the difference was confirmed by ANOVA at α = 0.05 level. Error bars indicate SD from triplicate experiments.

Using these overexpression lines and mutants, crude protein was extracted and glucosyltransferase activity toward IAA was analyzed using UDP-glucose as sugar donor. The data showed that overexpression lines with higher steady-state level of transcripts displayed higher enzyme activity toward IAA compared to wild type (Fig. 5A,B). However, the enzyme activity of mutants was not detected (data = 0). These results suggested that UGT74D1 activity toward IAA-glucose conjugation has been maintained *in planta*.

### UGT74D1 activity affected leaf positioning

**Figure 6:**
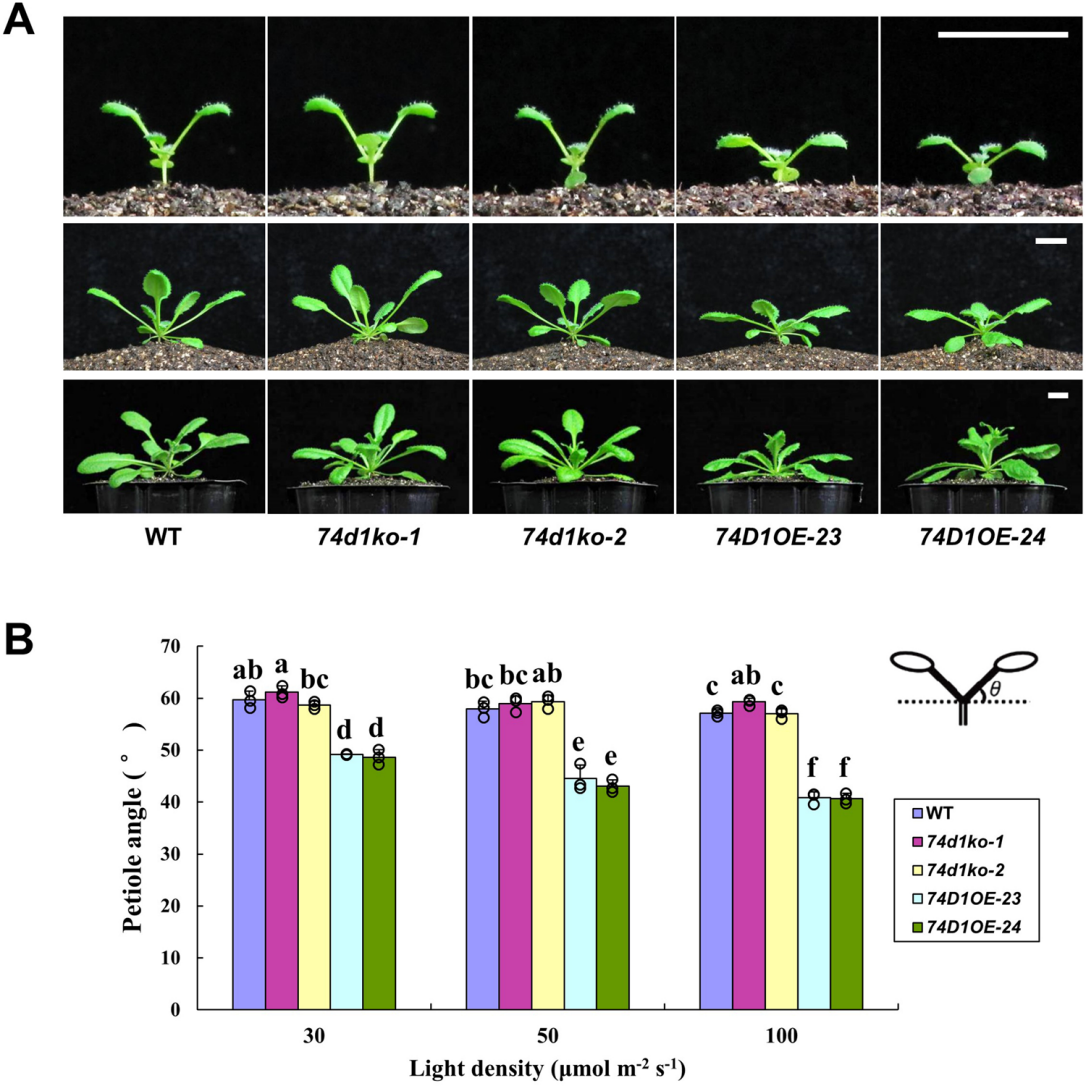
Leaf positioning phenotype of the transgenic lines. (**A**) Plants grown in soil for 2, 3 and 4 weeks, respectively. Scale bar = 1 cm. (**B**) Petiole angle of 2-week-old seedlings grown under different light intensities. At least 15 plants were tested within one replicate. The statistical significance of the difference was confirmed by ANOVA at α = 0.05 level. Error bars indicate SD from triplicate experiments.

Several previous studies reported that auxin glycosyltransferases could affect plant growth with curly leaves, compressed rosette, and shorter stature^14,15^. Here, UGT74D1 exhibited a distinct physiological relevance for leaves growth from other reported auxin glycosyltransferases. Although *UGT74D1* knock-out mutants did not show obvious phenotype possibly because of function redundancy, its overexpression lines displayed a clear change in leaf positioning (Fig. 6). The angle between the horizontal and the petiole of first true leaf was used as an index of leaf positioning and was measured. Our data showed that the petiole angle of *UGT74D1* overexpressor plants was much smaller compared with the wild-type (Fig. 6A). To test whether the leaf positioning can be influenced by the light intensity or not, transgenic plants were grown in 30 μmol m^−2^ s^−1^, 50 μmol m^−2^ s^−1^ and 100 μmol m^−2^ s^−1^, respectively. It was found that the leaf petiole angle of *UGT74D1* transgenic plants was always significantly smaller when compared to wild type and mutants in different light intensity (Fig. 6B). Because UGT74D1 was involved in glycosylation of IAA, this observation suggested that UGT74D1 might exert important influence on the petiole angle through changing auxin homeostasis.

### UGT74D1 altered auxin level and auxin distribution of leaf petioles

**Figure 7:**
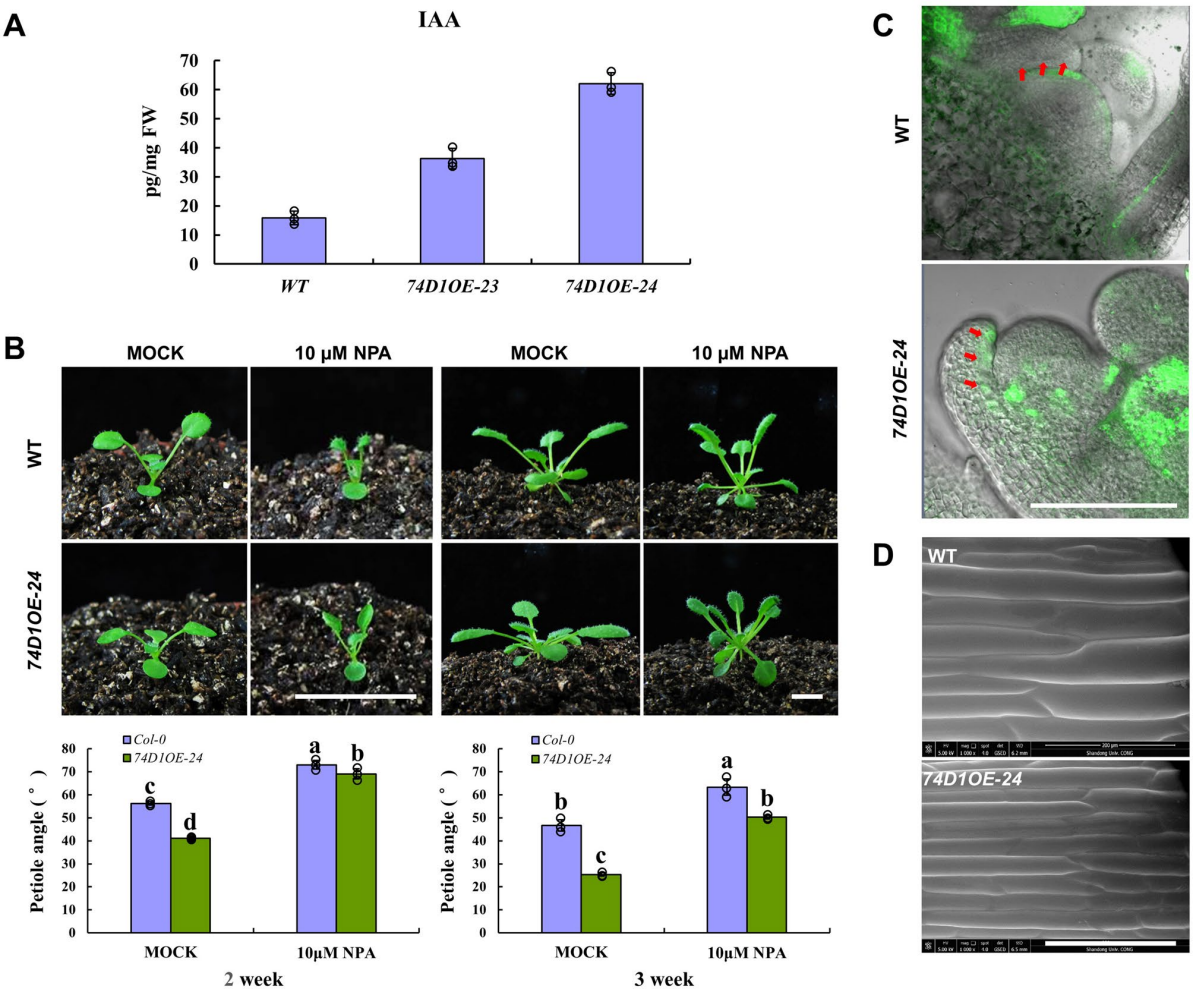
The analyses of IAA level and cell shape in leave petioles of *UGT74D1* OE lines. (**A**) The measurement of IAA level. (**B**) The response of leaf petiole angle to auxin transport inhibitor NPA. Scale bar = 1 cm. At least 15 plants were tested within one replicate. The statistical significance of the difference was confirmed by ANOVA at α = 0.05 level. Error bars indicate SD from triplicate experiments. (**C**) Auxin signal distribution indicated by GFP fluorescence in leaf primordia. Scale bar = 100 μm. (**D**) Cell shape of leaf petioles. Scale bar = 200 μm.

In order to know the possible influence of ectopically expressing UGT74D1 on the in vivo auxin level, the measurement of free auxins in leave petioles was performed. The most important native auxin, IAA, was detected in this research. As shown in Fig. 7A, it was found that the free form IAA was significantly increased compared to wild type, which suggests that auxin homeostasis was involved in leaf positioning and the IAA accumulation in leave petioles led to smaller petiole angle. To test this hypothesis, we used the polar auxin transport inhibitor NPA to chemically block auxin transport and disturb the auxin role. As shown in Fig. 7B, after treated with 10 μM NPA, both WT and *UGT74D1OE* exhibited increased leaf inclination compared with control (mock-treated). However, the petiole angle of overexpressors was still smaller than that of wild type, indicating a relative insensitivity of overexpressors to NPA because of increased IAA level.

To investigate the possible mechanism that resulted in the change of leaf petiole angle, we used the artificial auxin-responsive *DR5* promoter to monitor auxin signaling in leaf primordia^36,37^. Sections through shoot apices of Col-0/*pDR5::GFP* and *74D1OE*/*pDR5::GFP* plants were imaged by confocal microscopy. *Arabidopsis* wild type plants harboring a *pDR5::GFP* construct revealed low GFP signals in leaf primordia, however, strong GFP signals were detected on the adaxial side of leaf primordia of *UGT74D1OE* (Fig. 7C), indicating UGT74D1 might promote asymmetric auxin distribution and asymmetric cell growth on the adaxial zone of leaf primordia.

To test this hypothesis, the adaxial surface of leaf petiole was taken by scanning electron microscopy. As shown in Fig. 7D, cells on the adaxial side of *UGT74D1OE* leaf petiole were obvious longer compared to that of the WT, associated with decreased cell width. These observations suggested that the change of leaf petiole angle of *UGT74D1OE* was likely caused by the cell elongation on the adaxial surface of leaf petiole.

Previous studies have shown that one important aspect of the BR biological function is the regulation of leaf angle^18,19^. Accordingly, the sensitivities of *UGT74D1OE* lines to BR were examined. After *UGT74D1OE* lines were treated with 1 μM brassinolide (BL), one kind of active brassinosteroids, no obvious difference was found in leaf angle (Supplementary Fig. S1A). In addition, we analyzed whether *UGT74D1* transcription was induced by BR using the BR response factor *DWF4* as control. Our results indicated that *UGT74D1* transcription was not affected by BR (Supplementary Fig. S1B). Several BR related genes were also investigated for their expression level in *UGT74D1* transgenic plants, as shown in Supplementary Fig. S2, our results again indicated that relative expression level of BR related genes were not significantly changed in *UGT74D1* transgenic plants compared to WT. These data suggested that leaf positioning change caused by UGT74D1 is possibly independent of BR.

### UGT74D1 activity altered the expression of leaf growth related genes and auxin metabolic genes

**Figure 8:**
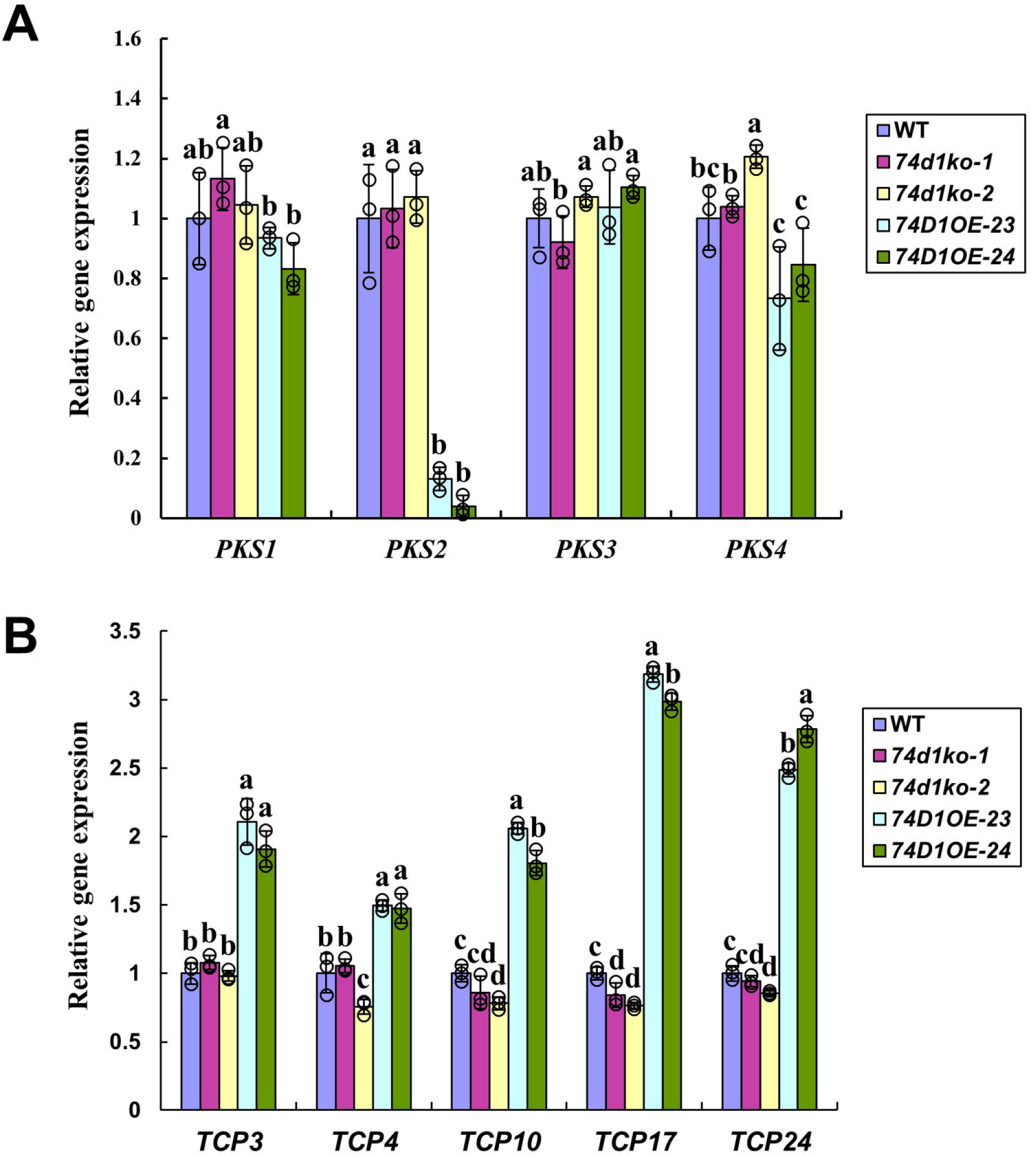
Altered expression level of leaf shape related genes. (**A**) Transcript level of *PKS* gene family. (**B**) Transcript level of *TCP* gene family. The relative transcript level was normalized to the transcript abundance of *Actin 2* gene. The statistical significance of the difference was confirmed by ANOVA at α = 0.05 level. Error bars indicate SD from triplicate experiments.

To further investigate the possible molecular mechanism leading to the leaf phenotype of *UGT74D1* transgenic plants, we analyzed the expression level of several key genes previously demonstrated to be involved in the leaf shaping and leaf positioning, including PKSs (*PKS1*, *PKS2*, *PKS3*, *PKS4*) and TCPs (*TCP3*, *TCP4*, *TCP10*, *TCP17*, *TCP24*)^38,39^. It was found that the *PKS2* transcript level was dramatically down-regulated in *UGT74D1* overexpression plants (Fig. 8A), which is in good agreement with the leaf angle phenotype. Moreover, the *TCP3*, *TCP10*, *TCP17* and *TCP24* were modulated to a significantly up-regulated expression level in *UGT74D1* overexpression lines (Fig. 8B).

**Figure 9:**
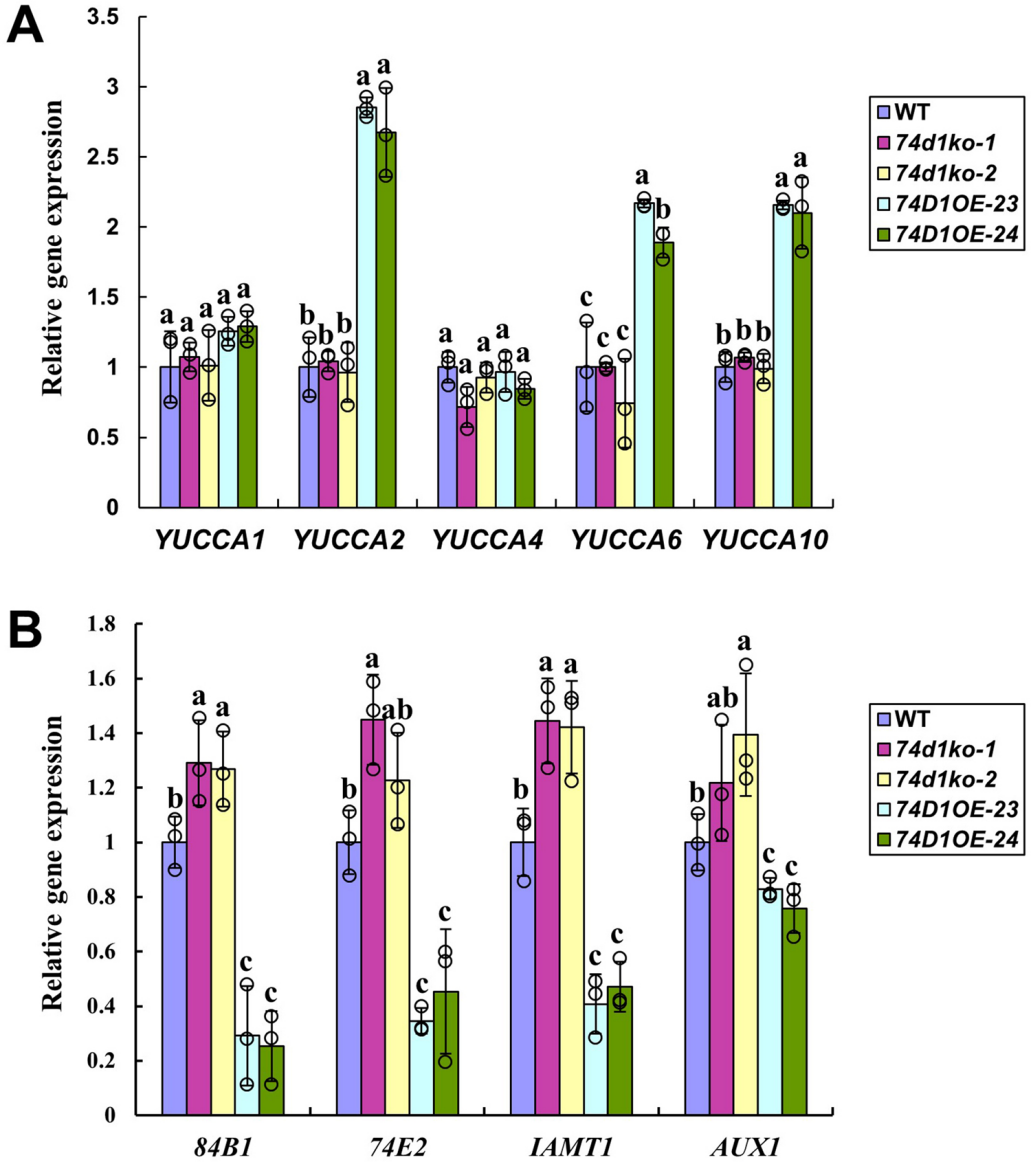
The change of expression level of genes involved in auxin metabolic pathways in *UGT74D1* overexpression lines and mutant lines. (**A**) Transcript level of *YUCs* genes. (**B**) Transcript level of *AUX1*, *UGT74E2*, *UGT84B1* and *IAMT1*. The relative transcript level was normalized to the transcript abundance of *Actin 2* gene. The statistical significance of the difference was confirmed by ANOVA at α = 0.05 level. Error bars indicate SD from triplicate experiments.

In addition, to know the reason causing auxin accumulation in *UGT74D1* overexpression lines, we tested the expression level of genes involved in auxin metabolic pathways. Although *ugt74d1* mutants did not show a clear change in expression level of auxin related genes, *UGT74D1* overexpression lines changed a lot in expression of several auxin related genes. The most important *YUC* genes that control IAA biosynthesis, including *YUCCA2*, *YUCCA6* and *YUCCA10*, were substantially up-regulated in overexpression lines, which was consistent with the accumulation of IAA in these transgenic plants (Fig. 9A). An auxin influx carrier, *AUX1*, was down-regulated in *UGT74D1* overexpression lines compared with wild-type, which might be a result of IAA accumulation (Fig. 9B). UGT84B1 and UGT74E2 were two different glucosyltransferases toward auxins identified previously^13–15^. We also investigated the expression of these two UGT genes. It was found that both *UGT84B1* and *UGT74E2* were down-regulated in *UGT74D1* overexpression lines, suggesting a functional redundancy of these auxin glucosyltransferases (Fig. 9B). *IAMT1* was found to be a gene encoding methyltransferase which converts IAA to methyl-IAA (MeIAA) and its overexpression in *Arabidopsis* leads to a curly leaf phenotype and perturbed auxin homeostasis^40^. Our analysis of *UGT74D1* transgenic plants indicated that *IAMT1* was significantly up-regulated in mutants but down-regulated in overexpression lines compared to wild type (Fig. 9B), implicating a link between glycosylation and methylation of auxins. Our data presented here suggested that the expression change of *UGT74D1* gene caused the expression change of many genes in auxin metabolic pathways and thus perturbed the auxin homeostasis.

## Discussion

Glycosyltransferase UGT74D1 catalyze the transfer of UDP-Glucose to IAA forming IAA-glucose. The free energy change of this reaction is positive and the energy of the acyl alkyl acetal bond between IAA and the aldehydic oxygen of glucose is above that of the phosphatogucose bond of UDPG^10,41^. This suggests that for the reaction to proceed, the level of UDPG must be significantly higher than that of IAA-glucose. Moreover, the accumulation of limited levels of IAA-glucose must be a second step of a transesterification. The energetics of these reactions makes it a candidate for regulatory control of IAA and IAA-glucose levels which can be looked at as a ‘buffer’ reaction. For example, the levels of IAA and 1-O-IAGlc track in parallel with each other in WT, low, medium and high UGT84B1 over-expression lines^14^. Similarly, a similar relationship between IAA and IAA-glucose levels was showed in ectopic maize IAGLU gene expression in *Arabidopsis*^42^. A similar approach using antisense showed a parallel decrease in free IAA and IAA-glucose in transgenic tomato^43^. In our research, IAA and IAA-glucose levels also showed a parallel increase in UGT74D1 over-expression *Arabidopsis* lines, which was consistent with these prior studies.

Hormone conjugation has been proposed to be significant contributors to hormone homeostasis. In plants, it is important to maintain appropriate hormone level in specific tissues and growth responses. In this research, an auxin-glucose conjugating enzyme, UGT74D1, was employed to explore the auxin homeostasis and the corresponding physiological responses *in planta*. We found that ectopically expressed *UGT74D1* caused obvious homeostasis alteration of auxins and changed leaf petiole positioning. However, *ugt74d1* mutants did not show clear changes in leaf growth and development. Why did *ugt74d1* mutants not display obvious phenotypic or physiological change? We supposed that other auxin glucosyltransferases compensated the loss of function in *ugt74d1* mutants. Besides UGT74D1, UGT84B1 and UGT74E2 were also identified as auxin glucosyltransferases of *Arabidopsis*^13–15^. Their physiological role might be partially overlapping, although ectopically expressed *UGT84B1* or *UGT74E2* gave a distinct phenotype^14,15^. In *UGT74D1* overexpression lines, we observed that the expression level of *UGT84B1* and *UGT74E2* were substantially down-regulated, which also suggested at least partial overlapping physiological roles between these three auxin glucosyltransferases. However, *UGT74D1* appears to have a distinct expression pattern. We observed that *UGT74D1* was expressed in whole leaves and petioles in developing young leaves, while it subsequently expressed in leaf margin as leaves grew and matured, suggesting that UGT74D1 may have different physiological effects from other auxin UGTs.

Recently, OxIAA was reported to be another substrate of UGT74D1 which converts OxIAA to OxIAA-Glc^44^. OxIAA is a primary IAA catabolite formed by IAA oxidation in *Arabidopsis*. It is inactive in bioassays and in auxin signaling^45^. Our data showed that *UGT74D1* overexpression lines had substantially increased free IAA. Even though UGT74D1 can catalyze the glucosylation of both IAA and OxIAA, the glucosylation of active auxin (IAA or its precursor IBA), rather than its inactive metabolite (OxIAA), might play direct and important role in modulating auxin homeostasis and leaf angle.

Our experimental results indicated that the transcriptional regulation of three *Arabidopsis YUCCA* genes, *YUCCA2*, *YUCCA6* and *YUCCA10*, was significantly enhanced in *UGT74D1* overexpression lines, which may be the reason leading to IAA accumulation to very high level in leaf petiole. However, the mechanism how glucosyltransferase UGT74D1 triggers the expression of YUCCAs is unclear. Several changes were also observed in other metabolic pathways and gene expressions. For example, overexpression of *UGT74D1* led to the down-regulated transcription of IAA influx carrier *AUX1*, IAA glucosyltransferases *UGT84B1* and *UGT74E2*, and IAA methyltransferase *IAMT1*. All these alterations may be a consequence of an increased IAA level in leaf petioles of *UGT74D1* overexpression plants.

Leaf positioning (petiole phototropism) is one of the important agronomic traits affecting plant architecture and yield. Also, leaf positioning is one of the adaptive processes in response to environmental light signals. Increasing evidences demonstrated the importance of PKS protein family in regulating leaf positioning. *PKS2* is found to be highly expressed in leaves^46,47^. The leaf position of the mutant *pks2* is found to have less erect petioles than wild-type. Moreover, auxin transport assays in mesophyll protoplast indicate that PKS2 may regulate light responses by regulating auxin homeostasis^26^. However, the link between PKS protein and auxin has not been firmly established during leaf development. In this study, we found that ectopically expressed UGT74D1 resulted in clear leaf angle change. When monitoring the IAA distribution, we found that IAA was concentrated on the adaxial side of leaf primordia in *UGT74D1OE* lines. Consistently with this observation, cells in the adaxial side of *UGT74D1OE* leaf petiole were obvious longer than wild type and the IAA level in leave petioles of *UGT74D1OE* lines was also higher than wild type. These observations might suggest that UGT74D1 could modulate auxin homeostasis and asymmetric distribution in leaves, thus altering leaf angle. Moreover, our data showed that the expression of *PKS2* was dramatically down-regulated in *UGT74D1* overexpression lines. These findings suggested the possibility that auxin homeostasis modulated by glucosyltransferase UGT74D1 could provide feedback to PKS2 expression and then influence the leaf positioning in *Arabidopsis*.

TCP family transcription factors are among the best-characterized regulators of leaf development^27,28^. In *Arabidopsis*, there were 13 class I TCPs and 11 class II TCPs^38^. TCPs play an essential role in the determination of leaf size and shape by regulating cell proliferation and differentiation. Besides, it is suggested that TCPs control leaf shape by promoting leaf maturation in a threshold activity manner^39^. Recently, a novel transcriptional repressor EAR motif protein1 (TIE1) which contain a TCP interactor was demonstrated to be a major modulator of TCP activities during leaf development^48^. It was supposed that the interaction of TIE1 and TCPs regulates the expression of auxin related genes and controls cell differentiation and leaf development^48^. In this research, we found that the ectopic expression of UGT74D1 led to a significant up-regulation for the transcription of *TCP3*, *TCP10*, *TCP17* and *TCP24*. We supposed that a feedback circle between auxin pathway modulated by UGT74D1 glucosyltransferase and TCP pathway may exist. Considering the involvement of both PKS2 and several TCPs in the leaf positioning, our findings suggested that UGT74D1 represents a potentially unique paradigm in the regulation of leaf angle in *Arabidopsis*. However, the possible link between PKS protein and TCP protein remains to be answered in the case of leaf positioning.

## Materials and methods

### Plant material and growth conditions

All the *Arabidopsis thaliana* plants used in this work were of the Col-0 ecotype. The two T-DNA insertion mutants (*74d1ko-1*, Salk_004870; *74d1ko-2*, Salk_011286) and the four *UGT74D1* overexpression lines (*74D1OE-11*, *-23*, *-24*, *-26*) used in this research are consistent with the same plant lines used in previous article by Jin et al.^29^. The *Arabidopsis thaliana pDR5::GUS* and *pDR5::GFP* seeds were provided from Dr. Zhaojun Ding, Shandong University, Qingdao, China. Plants were grown on Nutrition Soil with vermiculite (Nutrition Soil:vermiculite, 2:1) or Murashige and Skoog (MS) basal medium plates containing 3% (w/v) sucrose and 0.7% (w/v) agar. The conditions for growing room were set at 22 ± 2 °C with a light intensity of 100 μmol m^−2^ s^−1^. Light regime is controlled at 16 h of light and 8 h of darkness.

### Construction of *UGT74D1 promoter::GUS* and histochemical GUS assays

*UGT74D1* (*AT2G31750*) promoter was amplified from *Arabidopsis* genomic DNA with the primers 5′-CCCAAGCTTGCAATTGGGGTTTCATGCTTAC-3′ and 5′-CGGGATCCGCTTTCGCTTTCTCTCCCATTG-3′ and a 2 kb DNA fragment upstream of start codon was obtained. The *Bam*H1-*Hin*dIII digested *UGT74D1* promoter fragment was sub-cloned into the pBI121 vector to replace the cauliflower mosaic virus (CaMV) 35S promoter and yield the *UGT74D1 promoter::GUS* fusion construct. The fusion vector was transferred into *Arabidopsis* plants through the floral dip method^49^, and homozygous plants were subjected to GUS staining according to the method of Jefferson^50^.

### Crude protein extraction and glucosyltransferase assay

Crude protein was extracted from 2-week-old transgenic seedlings according to Jackson et al.^14^. To investigate the glycosyltransferase activity of the crude protein extracts prepared from plant tissues, 50 mL crude protein extracts were incubated at 37 °C for 1 h according to Jin et al.^29^. The reaction mix was analyzed subsequently using reverse-phase HPLC following the method described by Jin et al.^29^.

### Analysis of free IAA in leaf petioles

Leaf petioles of the 10-day-old seedlings of wild type Arabidopsis thaliana Col-0 and two UGT74D1 overexpressing lines (74D1OE-23, 74D1OE-24) were used for analysis of free IAA level. 2 cm proximal end of leaf petioles were collected in five replicates, weighed, immediately frozen in liquid nitrogen and stored at − 80 °C until extraction. Then frozen samples were ground in liquid nitrogen with mortar and pestle. IAA quantification was determined on ultra high performance liquid chromatography-triple quadrupole mass spectrometry (UPLC-MS/MS) with negative electrospray ionization mode and 100 pmol isotope-labeled ^2^H_2_-IAA served as the internal standard as described by Fu et al.^51^.

### Total RNA extraction and quantitative RT-PCR (qRT-PCR)

To study the expression level of leaf development related genes, 2-week-old seedlings were harvested for RNA extraction. For investigating whether *UGT74D1* gene was induced by IAA and IBA, 2-week-old seedlings was first soaked with 10 μM IAA and IBA, respectively, for 0–24 h, then they were harvested for RNA extraction. Total RNA was extracted using Trizol reagent and was used as template for cDNA synthesis. The relative transcript level was normalized with *Actin 2* gene according to the 2^−ΔΔCT^ method^52^.

### Microscopy imaging

For the fluorescence images, LSM 700 confocal laser scanning microscope was used. Images were obtained and processed using the ZEN 2009 software. For subcellular localization analysis, the *UGT74D1* open reading frame without stop codon was amplified, and then inserted into p326-SGFP vector to generate the 74D1-GFP fusion gene driven by CaMV35S promoter^53,54^. 74D1-GFP fusion plasmid was transformed into *Arabidopsis* to get transgenic plants. The roots of the 5-day-old transgenic seedlings were detected using a confocal laser-scanning microscope at excitation wavelengths of 488 and 647 nm, respectively. Counterstaining of cell walls was achieved by mounting seedling roots in 10 μM propidium iodide.

For auxin signaling analysis of shoot apices, the Col-0 seedlings harboring *pDR5::GFP* construct driven by CaMV35S promoter was cross-fertilized with wild-type and *UGT74D1OE*, respectively. The F1 generation was harvested to generate the heterozygous plants. Then *Arabidopsis* vegetative shoot apices were separated by removing older leaves and fluorescence signals were monitored directly using a confocal laser-scanning microscope.

Environmental scanning electron microscopy was performed for the cellular observation of leaf petioles. The basis of leaf petiole (1 cm long) was excised from first pair of true leaves (14-day-old plants) after second pair of true leaves emerged. Tissue was then transferred to peltier cooling stage (temperature setting = 5 °C). Precooled metal stubs with the samples were transferred to the cooling stage and images were recorded.

## Supplementary Information

Supplementary Legends.

Supplementary Figure S1

Supplementary Figure S2.
