# Supplementary material for: The ectopic expression of Arabidopsis glucosyltransferase UGT74D1 affects leaf positioning through modulating indole-3-acetic acid homeostasis
Source: Sci Rep. 2021 Jan 13;11:1154. doi: 10.1038/s41598-021-81016-x (PMC7806859; doi:10.1038/s41598-021-81016-x)
Supplement: Supplementary file 1 — Supplementary Legends. [file 41598_2021_81016_MOESM1_ESM.docx]

**Supplementary information**

**Figure S1.** The leaf positioning phenotype of the transgenic lines was not affected by BR. (**A**) The leaf positioning phenotype of wild type and *UGT74D1OE* seedlings treated by 1 μM BL. Scale bar = 1 cm. (**B**) BR induction analysis of *UGT74D1* transcription using the BR response factor *DWF4* as the control. The relative transcript level was normalized to the transcript abundance of *Actin 2* gene. The statistical significance of the difference was confirmed by ANOVA at α = 0.05 level. Error bars indicate SD from triplicate experiments.

**Figure S2.** Expression level of BR-related genes in wild type, *ugt74d1* mutants and *UGT74D1OE* lines. The relative transcript level was normalized to the transcript abundance of *Actin 2* gene. The statistical significance of the difference was confirmed by ANOVA at α = 0.05 level. Error bars indicate SD from triplicate experiments.
