# Supplementary figures and images for: The ectopic expression of Arabidopsis glucosyltransferase UGT74D1 affects leaf positioning through modulating indole-3-acetic acid homeostasis
Source: Sci Rep. 2021 Jan 13;11:1154. doi: 10.1038/s41598-021-81016-x (PMC7806859; doi:10.1038/s41598-021-81016-x)

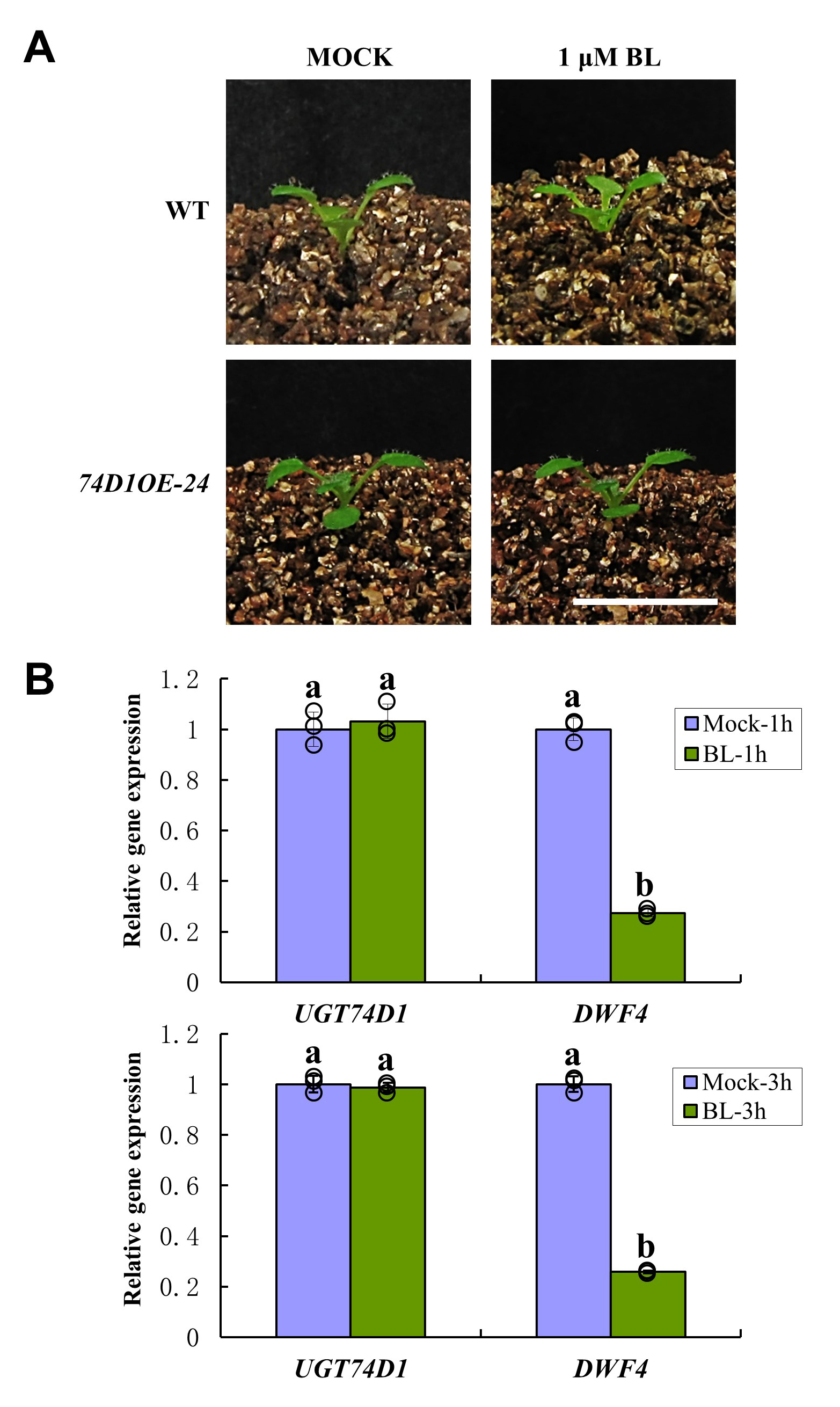

Supplement: Supplementary file 2 — Supplementary Figure S1 [file 41598_2021_81016_MOESM2_ESM.jpg]

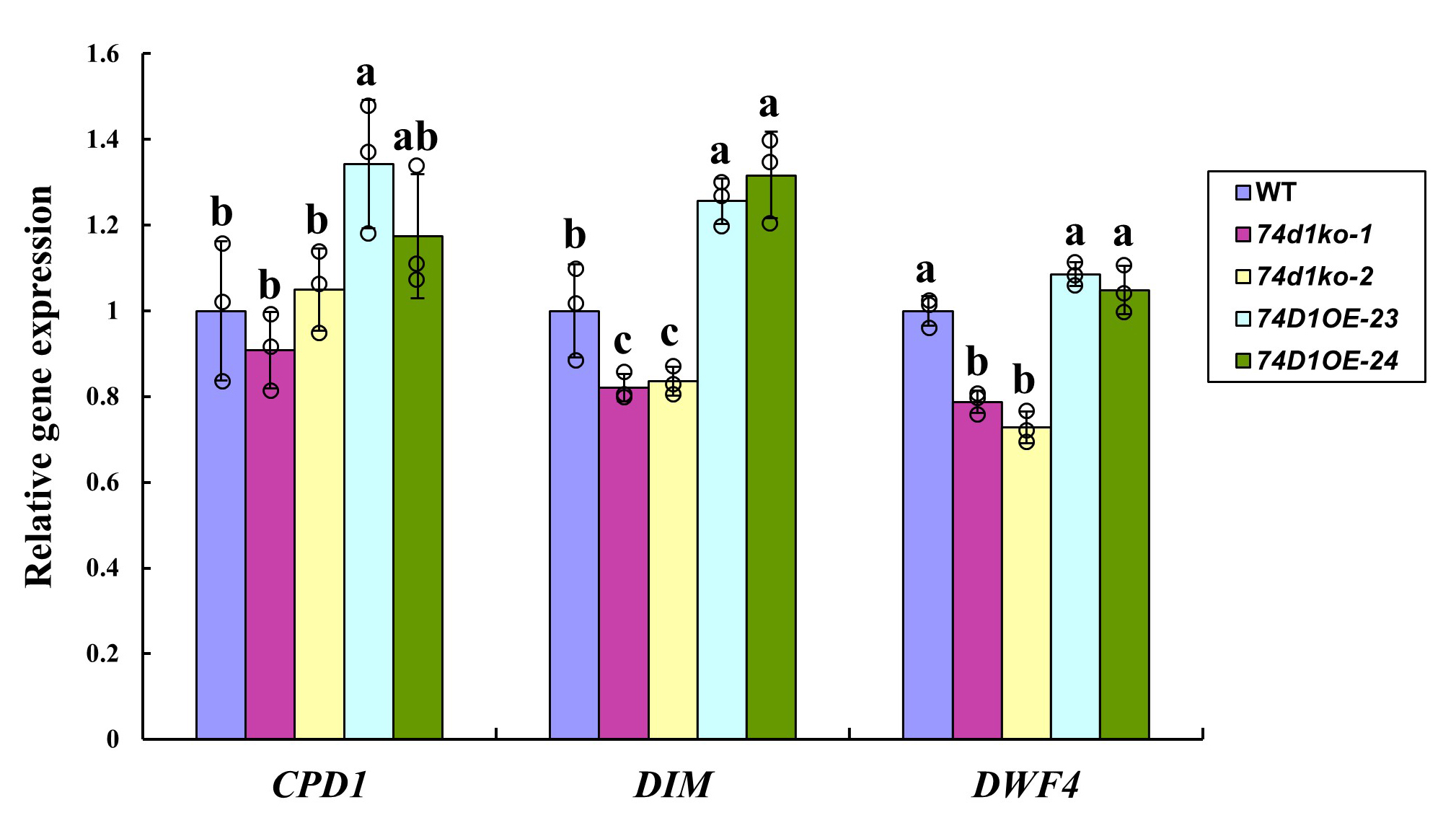

Supplement: Supplementary file 3 — Supplementary Figure S2. [file 41598_2021_81016_MOESM3_ESM.jpg]
